# Quality of life in anorexia nervosa, bulimia nervosa and eating disorder not-otherwise-specified

**DOI:** 10.1186/2050-2974-1-43

**Published:** 2013-11-26

**Authors:** Hannah DeJong, Anna Oldershaw, Lot Sternheim, Nelum Samarawickrema, Martha D Kenyon, Hannah Broadbent, Anna Lavender, Helen Startup, Janet Treasure, Ulrike Schmidt

**Affiliations:** 1 Department of Psychological Medicine, Section of Eating Disorders, King’s College London, Institute of Psychiatry, De Crespigny Park, PO 59, London SE5 8AF, England; 2 South London and Maudsley NHS Foundation Trust, London, UK

**Keywords:** Eating disorders, Anorexia nervosa, Bulimia nervosa, Clinical impairment assessment, Functional impairment, Quality of life

## Abstract

**Background:**

This study aimed to assess differences in Quality of Life (QoL) across eating disorder (ED) diagnoses, and to examine the relationship of QoL to specific clinical features.

**Results:**

199 patients with a diagnosed ED completed the Clinical Impairment Assessment (CIA) [*Cognitive Behavior Therapy and Eating Disorders*, 315–318, 2008] and the Eating Disorders Examination (EDE) [*Int J Eat Disord* 6:1–8]. Differences between diagnostic groups were examined, as were differences between restrictive and binge-purge subtypes.

CIA scores and EDE scores were positively correlated and higher in groups with binge-purge behaviours. CIA scores were not correlated with BMI, illness duration or frequency of bingeing/purging behaviours, except in the binge-purge AN group, where CIA scores negatively correlated with BMI.

**Conclusions:**

Patients with EDs have poor QoL and impairment increases with illness severity. Patients with binge/purge diagnoses are particularly impaired. It remains unclear which clinical features best predict the degree of impairment experienced by patients with EDs.

## Background

In recent years, the assessment of Quality of Life (QoL) in people with eating disorders (EDs) has been subject to considerable research interest. The World Health Organisation defines QoL as ‘an individual’s perception of their position in life in the context of the culture and value system in which they live and in relation to their goals, expectations, standards and concerns’ [1]; page 145. It is a multidimensional construct that is understood to include perceptions about various areas of life, including physical, psychological, social and emotional domains [1]. QoL measures are beginning to be recognized as a key patient-oriented measure of outcome [2]. To date, studies in EDs have indicated significantly reduced QoL in this patient group, to a degree that is comparable with QoL findings in various other serious illnesses and disorders, such as angina and anxiety disorders [3,4]. Individuals with EDs seem to be particularly impaired in psychological and social domains [5,6].

Patterns of diagnostic differences are inconsistent across studies, with some studies finding no significant differences between diagnostic groups [7,8], some reporting lower QoL in individuals with anorexia nervosa (AN) than bulimia nervosa (BN) [9-11], and others reporting higher QoL in AN [12,13]. However, studies that report higher QoL in AN also report seemingly contradictory findings, such as an increased presence of suicidal thoughts and self-harming behaviours in this group [12]. It is also notable that studies finding less impairment in AN have tended to use generic, rather than ED specific measures of QoL, which - as has been hypothesized - may be less sensitive to certain aspects of impairment associated with these disorders [14]. Inconsistencies in the differences reported across diagnostic groups may also be attributable to the presence of diagnostic subtypes. There is some evidence that individuals with bingeing and/or purging forms of AN are more impaired than individuals with restrictive AN [8,15]. Several authors have suggested that a lack of insight into the negative impact of restrictive AN may inflate self-reported measures of QoL in this group – restrictive EDs are often experienced as ego-syntonic due to the valued weight loss associated with these disorders [13,16].

It remains unclear how specific symptoms and behaviours associated with EDs impact on QoL. There is evidence that the presence of both bingeing [9,17-19] and purging behaviours [14,20,21] negatively affect QoL in both clinical and community samples, but evidence regarding the impact of the frequency of these behaviours is sparse and contradictory [6,19,22,23]. The effect of BMI on QoL is relatively unexplored, particularly in underweight ED samples [6], though there is some evidence that QoL falls with decreased BMI [11]. There is similarly little evidence on the relationship between illness chronicity and QoL, though one study has found lower QoL in individuals with illness duration greater than five years compared to those ill for less than five years [24].

The present study used the Clinical Impairment Assessment (CIA) [25,26], a measure of QoL specifically designed for use in ED populations. The CIA is designed to assess the perceived effects of having an ED on various domains, including social, emotional and cognitive aspects. Relevant domains were identified by clinicians and through interviews with patients diagnosed with an ED. The authors decided not to include items about possible physical effects of having an ED (e.g. feeling faint or cold, palpitations), arguing that individuals with EDs may not necessarily associate these effects with their eating behaviours. The CIA specifically assesses impairment that occurs *as a result of an ED*, whether stemming from eating behaviours or associated concerns about body shape. CIA scores have been shown to correlate closely with clinician ratings of psychosocial impairment, suggesting that the measure accurately captures clinically relevant information [26]. CIA scores have also been shown to correlate with ED symptom severity [26], as measured by the Eating Disorder Examination Questionnaire (EDE-Q) [27]. This supports the authors’ assertion that the CIA specifically measures impairment that is perceived as secondary to (that is it results from) an individual’s ED.

The aims of the current study were to extend previous findings with the CIA, using a large ED sample composed of a mixture of diagnoses. In particular, the study sought to increase the available data on CIA scores in individuals with AN –previous work with this measure has included only small numbers of participants with this diagnosis [26,28] – and to consider possible differences between restrictive and binge-purge subtypes. In addition, the study aimed to examine the relationships between CIA score and clinical features including Eating Disorder Examination scores [29], BMI, illness duration and diagnosis. We hypothesised that individuals with a diagnosis of AN would report more impairment on the CIA than individuals with BN or EDNOS, as previous findings indicate that people with AN often report more impairment than other groups when ED-specific measures of QoL are used [11,30]. We expected to replicate findings [26] of a positive correlation between CIA scores and ED severity, as measured by the EDE. We also hypothesised that CIA scores would be higher in patients with a longer duration of illness, in patients with lower BMIs and in patients with more frequent bingeing and purging behaviours.

## Methods

### Participants

Participants were all treatment-seeking individuals who were assessed at an outpatient ED service and recruited to take part in three clinical trials of outpatient treatments for anorexia and bulimia nervosa, recruiting from catchment area based specialist ED services and with minimal exclusion criteria [31-33]. The data reported here were collected at baseline, as part of a larger assessment battery used prior to randomising participants to a treatment condition. Participants were primarily recruited from the outpatient ED service of the South London and Maudsley NHS Foundation Trust. A small number of participants (N = 4) were recruited from other sites within London. Ethical approval for each trial was granted by a local NHS Research Ethics Committee.

Exclusion criteria were insufficient literacy or English language to complete research assessments, presence of serious physical or psychiatric co-morbidity requiring treatment in its own right (e.g. substance dependence, psychosis, diabetes), history of head injury or current pregnancy. Participants taking antidepressant medications were included, provided their dose had remained stable over the preceding four weeks.

The final sample reported here included 199 patients (N = 189 female) with a DSM-IV-TR [34] diagnosis of either AN (N = 84), BN (N = 49) or EDNOS (N = 66). Diagnoses were established by experienced ED clinicians during an initial clinical assessment. Due to the heterogeneity of AN and EDNOS as diagnoses, patients in these groups were also classified as having a restrictive presentation or a binge-purge presentation, as defined in [31-33]. The AN group was composed of 42 restrictive and 42 binge-purge cases; the EDNOS group was composed of 28 restrictive and 38 binge-purge cases.

A power calculation revealed that at a total sample size of 199 across the 5 groups, distributed across the groups as specified, a one-way analysis of variance will have 90% power to detect at the 0.01 level an effect size of 0.11.

### Measures

Diagnosis and duration of illness were established during an initial clinical assessment. The measures described below were then administered by a researcher, as part of a larger battery of measures associated with one of the clinical trials. Height and weight were also measured during this assessment and used to calculate BMI (kg/m^2^).

#### Clinical impairment assessment (CIA)

The CIA (25,26) is a 16-item measure of functional impairment designed for use in individuals with EDs. It begins with the stem question: “Over the past 28 days, to what extent have your eating habits, exercising or feelings about your eating, shape and weight…?”. This question is followed by 16 items enquiring about different types of impairment (e.g. “… made it difficult to concentrate?”; “…made it difficult to eat out with others?”). Each item is rated on a 4-point scale from ‘not at all’ (scored as 0) to ‘a lot’ (scored as 3). Some items are marked ‘if applicable’ and can be left blank if not relevant for the participant.

The CIA is scored by adding all items to give a total score. Any missing items are pro-rated and added to this total, provided that at least 12 of the 16 items have been rated. The CIA therefore generates a single global score, with higher values indicating greater functional impairment. The maximum possible score is 48. The CIA has been shown to have satisfactory internal consistency, test-retest reliability, construct and discriminant validity and also sensitivity to change [25].

#### Eating disorder examination (EDE)

The EDE (29) is a semi-structured interview designed to assess key behaviours and cognitions associated with ED psychopathology. In common with the CIA, it focuses on the preceding 28 days. Each item of the interview is numerically scored and then used to generate four subscale scores (dietary restraint, eating concern, weight concern and shape concern). The mean of these subscale scores can also be used as a global measure of ED psychopathology. The range of possible scores in each case is 0 to 6, with higher scores indicating greater severity.

The EDE also gathers information on key ED behaviours over the preceding 28 days, including the number of objective binge episodes (OBEs), number of subjective binge episodes (SBEs) and number of episodes of self-induced vomiting. OBEs are defined as episodes in which the quantity of food eaten is objectively large and the individual experiences a loss of control while eating. In contrast, SBEs are defined as episodes in which the individual experiences a loss of control and views the amount eaten as excessive, but the amount eaten is not objectively large.

The EDE is a reliable and widely used assessment of ED symptomatology with good discriminant validity and satisfactory internal reliability [35].

### Analysis

All demographic and clinical variables were non-normally distributed and attempts to transform these variables were unsuccessful. Non-parametric analyses were therefore used and group differences examined using Kruskall Wallis tests, followed by post-hoc Mann Whitney *U* tests.

The distribution of the CIA scores was also non-normal. Outliers were defined as values lying more than 2.5 standard deviations from the mean, and were removed from further analyses (N = 4). CIA scores remained non-normally distributed and attempts to transform the scores were unsuccessful. Non-parametric analyses were therefore used to examine group differences in CIA scores. Kruskall Wallis tests were conducted, followed by post-hoc Mann Whitney *U* tests. Effect sizes were calculated using the formula *r* = Z/√N, then converted to Cohen’s *d* scores. Effect sizes are defined as small (*d* < 0.4), medium (0.4 ≤ *d* < 0.8) or large (*d* ≥ 0.8).

The AN and EDNOS groups were then divided into restrictive (R) and binge-purge (BP) subgroups, creating a total of five diagnostic groups (AN-R, AN-BP, EDNOS-R, EDNOS-BP, BN). Differences in demographic and clinical variables were examined using Kruskall Wallis tests and post-hoc Mann Whitney *U* tests. Differences in CIA scores across these groups were examined in the same way, and effect sizes calculated as described above.

Correlations between CIA scores and clinical variables were examined using Spearman’s rank correlation analyses. Analyses were conducted for the sample as a whole, and also separately for each diagnostic group and subgroup. In all cases a significance level of α = .05 was used.

## Results

### Demographic and clinical characteristics: AN, BN, EDNOS

As Table 1 shows, the three diagnostic groups did not differ in age, duration of illness, global EDE score, eating concern subscale scores or weight concern subscale scores. Group differences in BMI were as expected – BMI was lowest in the AN group, highest in the BN group and intermediate in the EDNOS group. The AN group also reported higher dietary restraint and lower shape concern than the BN and EDNOS groups, who did not differ from one another. The BN group reported more OBEs and more episodes of self-induced vomiting than the AN and EDNOS groups, who did not differ from one another. The BN group also reported more SBEs than the AN group, with the EDNOS group not significantly different from AN or BN.

**Table 1:** Demographic and clinical characteristics: AN, BN, EDNOS

	**Median (IQR)**				
	**AN (n** = **84)**	**BN (n** = **49)**	**EDNOS (n** = **66)**	**Kruskall Wallis statistic**	**Post-hoc tests**
**Age (yrs)**	24.50 (10.0)	28.00 (9.00)	26.50 (10.25)	n.s.	
**Duration of illness (yrs)**	7.00 (8.50)	9.00 (10.25)	9.00 (9.00)	n.s.	
**BMI**	16.30 (1.78)	22.00 (6.34)	18.40 (5.45)	*H* = 100.97 p < .001	AN < EDNOS < BN
**EDE global score**	3.40 (1.83)	3.79 (1.06)	3.62 (2.14)	n.s.	
**EDE dietary restraint**	3.90 (2.05)	3.60 (1.87)	3.20 (2.75)	*H* = 9.13 p = .010	AN > BN = EDNOS
**EDE eating concern**	2.90 (2.00)	3.00 (2.20)	2.70 (2.35)	n.s.	
**EDE weight concern**	3.25 (2.35)	4.43 (1.94)	4.35 (2.60)	n.s.	
**EDE shape concern**	3.60 (2.15)	5.07 (1.32)	4.48 (2.58)	*H* = 23.06 p < .001	AN < EDNOS = BN
**OBE**	0.00 (4.50)	16.00 (22.50)	0.00 (9.75)	*H* = 46.66 p < .001	AN = EDNOS < BN
**SBE**	0.00 (7.00)	6.00 (19.00)	1.00 (16.00)	*H* = 6.78 p = .034	AN < BN
**Self induced vomiting**	0.00 (12.25)	15.00 (23.50)	0.00 (11.50)	*H* = 24.917 p < .001	AN = EDNOS < BN

Notes: AN, anorexia nervosa, BN, bulimia nervosa, EDNOS, eating disorder not-otherwise-specified, BMI, Body Mass Index, EDE, Eating Disorder Examination, OBE, Objective Binge Episodes, SBE, Subjective Binge Episodes.

The groups were therefore broadly similar in terms of age, illness duration and illness severity, with some differences in specific features that seemed consistent with the diagnostic profile of each group.

### CIA scores: AN, BN, EDNOS

There was no significant difference between the AN, BN and EDNOS groups in CIA scores [*H*(2) = 3.96, *p* = .138]. The median scores for each group were: AN median = 34.0, IQR = 13.1; BN median = 34.13, IQR = 8.23; EDNOS median = 31.0, IQR = 13.68.

### Demographic and clinical characteristics: restrictive and binge-purge subtypes

As Table 2 shows, the five diagnostic subgroups did not differ in age or duration of illness, but did differ in BMI, EDE global and subscale scores and frequency of ED behaviours. In the case of BMI, the AN-R group had the lowest BMIs, the AN-BP and EDNOS-R groups had intermediate BMIs, and the EDNOS-BP and BN groups had the highest BMIs. In general, individuals with binge-purge spectrum EDs (that is BN, AN-BP, EDNOS-BP) had higher EDE scores than individuals with restrictive EDs and more frequently engaged in bingeing and purging behaviours. A somewhat different pattern emerged for the dietary restraint subscale of the EDE, for which the AN-BP group scored higher than all other groups, who did not differ from one another. Group differences therefore were broadly in line with the diagnostic profile of each group, and suggest a somewhat greater illness severity in the binge-purge spectrum groups.

**Table 2:** Demographic and clinical characteristics: restrictive and binge-purge subtypes

	**Median (IQR)**						
	**AN-R (n** = **42)**	**EDNOS-R (n** = **28)**	**AN-BP (n** = **42)**	**EDNOS-BP (n** = **38)**	**BN (n** = **49)**	**Kruskall Wallis**	**Post hoc**
**Age**	23.0 (10.5)	26.0 (12.0)	25.0 (9.0)	27.0 (8.0)	28.0 (9.0)	n.s.	
**Duration of illness**	7.0 (7.13)	6.0 (8.0)	7.0 (12.0)	9.0 (9.25)	9.0 (10.25)	n.s.	
**BMI**	16.0 (2.33)	17.2 (2.2)	16.8 (1.65)	22.4 (6.47)	22.0 (6.34)	*H* = 127.08, p < .001	ANR < ANBP = EDNOSR < BN = EDNOSBP
**EDE global score**	2.95 (1.85)	2.7 (2.5)	3.9 (1.75)	4.06 (1.49)	3.79 (1.06)	*H* = 18.03, p = .001	EDNOSR = ANR < BN = ANBP = EDNOSBP
**EDE dietary restraint**	3.65 (2.35)	3.2 (3.4)	4.4 (1.7)	3.0 (2.6)	3.6 (1.87)	*H* = 16.18, p = .003	EDNOSBP = EDNOSR = BN = ANR < ANBP
**EDE eating concern**	2.4 (2.75)	1.6 (4.0)	3.3 (2.0)	3.2 (1.7)	3.0 (2.2)	*H* = 16.52, p = .002	EDNOSR = ANR < BN = EDNOSBP = ANBP
**EDE weight concern**	3.2 (2.65)	3.5 (3.38)	3.35 (2.5)	4.6 (1.43)	4.43 (1.94)	*H* = 16.87, p = .002	ANR = ANBP = EDNOSR < BN = EDNOSBP
**EDE shape concern**	3.6 (2.48)	2.8 (3.1)	3.85 (2.1)	5.07 (1.22)	5.07 (1.32)	*H* = 47.39, p < .001	EDNOSR = ANR = ANBP < EDNOSBP = BN
**OBE**	0.0 (0.0)	0.0 (0.0)	3.0 (20.0)	5.0 (27.0)	16.0 (22.5)	*H* = 93.74 p < .001	ANR = EDNOSR < ANBP = EDNOSBP < BN
**SBE**	0.0 (2.0)	0.0 (1.0)	2.0 (14.5)	8.0 (23.5)	6.0 (19.0)	*H* = 24.91 p < .001	ANR = EDNOSR < ANBP = BN = EDNOSBP
**Self induced vomiting**	0.0 (0.0)	0.0 (0.0)	9.0 (28.0)	6.0 (18.0)	15.0 (23.5)	*H* = 70.26 p < .001	ANR = EDNOSR < EDNOSBP < BN EDNOSBP = ANBP = BN

Notes: AN, anorexia nervosa, BN, bulimia nervosa, EDNOS, eating disorder not-otherwise-specified, R, restrictive, BP, binge-purge, BMI, Body Mass Index, EDE, Eating Disorder Examination, OBE, Objective Binge Episodes, SBE, Subjective Binge Episodes.

### CIA scores: restrictive and binge-purge subtypes

There was a significant difference in CIA scores across the diagnostic subgroups [*H*(4) = 9.49, *p* = .05] (for medians and IQRs see Table 3). Post-hoc tests indicated that the AN-BP group had higher CIA scores than both the AN-R [*U* = 628.0, *p* = .049, *d* = 0.45] and EDNOS-R [*U* = 362.5, *p* = .012, *d* = 0.64] groups, who did not differ from one another. The BN group also had higher CIA scores than the EDNOS-R group [*U* = 464.0, *p* = .032, *d* = 0.51]. All group differences were of medium effect size.

**Table 3:** Median CIA scores for restrictive and binge-purge subtypes

**Diagnostic subtype**	**n**	**Median CIA score (IQR)**
AN-R	42	31.0 (11.0)
EDNOS-R	28	28.0 (17.27)
AN-BP	42	36.5 (15.58)
EDNOS-BP	38	32.0 (12.73)
BN	49	34.1 (8.23)

Notes: AN, anorexia nervosa, BN, bulimia nervosa, EDNOS, eating disorder not-otherwise-specified, R, restrictive, BP, binge-purge, CIA, Clinical Impairment Assessment Questionnaire.

### Correlations between clinical variables and CIA scores

#### Whole sample

CIA scores were correlated with the EDE global score [*r* = .44, *p* < .001], and with all EDE subscale scores – dietary restraint [*r* = .27, *p* < .001], eating concern [*r* = .48, *p* < .001], weight concern [*r* = .39, *p* < .001], and shape concern [*r* = .33, *p* < .001]. CIA scores were not correlated with duration of illness, BMI, objective or subjective binge frequency, or frequency of self-induced vomiting.

#### Diagnostic groups

In the AN and BN groups, CIA scores were positively correlated with EDE global score and all subscales except dietary restraint. In the EDNOS group, CIA scores were positively correlated with EDE global score and all subscale scores (see Table 4). CIA scores were not correlated with duration of illness, BMI or frequency of binge-purge behaviours in any of the diagnostic groups.

**Table 4:** Correlations between CIA and EDE scores

		**Correlation with CIA score**				
		**EDE global**	**EDE dietary restraint**	**EDE eating concern**	**EDE weight concern**	**EDE shape concern**
Diagnostic group	AN	.407**	.213	.403**	.435**	.306**
	BN	.456**	.071	.514**	.493**	.384**
	EDNOS	.479**	.419**	.516**	.361**	.427**
Diagnostic subtype	AN-R	.429**	.187	.276	.500**	.475**
	EDNOS-R	.571**	.464*	.547**	.396*	.539**
	AN-BP	.321*	.138	.396**	.366*	.164
	EDNOS-BP	.436**	.417*	.514**	.284	.317
	BN	.456**	.071	.514**	.493**	.384**

Notes: **p < .01, *p < .05, AN, anorexia nervosa, BN, bulimia nervosa, EDNOS, eating disorder not-otherwise-specified, R, restrictive, BP, binge-purge, CIA, Clinical Impairment Assessment Questionnaire, EDE, Eating Disorder Examination.

#### Restrictive/binge-purge subgroups

Across the five diagnostic subgroups, CIA scores were positively correlated with EDE global scores, and with many of the subscale scores (see Table 4). In the AN-R, EDNOS-R, EDNOS-BP and BN groups, there were no correlations with any other clinical variables. However, in the AN-BP group there was a negative correlation between CIA score and BMI [*r* = −.43, *p* = .005] and a near-significant correlation between CIA score and objective binge frequency [*r* = −.30, *p* = .054].

## Discussion

In the present sample, all ED groups had very elevated CIA scores compared to previously reported population norms: Young adult women in Sweden: M = 8.3, SD = 9.4 [28], adolescent Fijian girls: M = 11.6, SD = 10.9) [36], young adult female Norwegian University students; M = 6.4, SD = 7.5 [37]. There were no differences in CIA scores between diagnostic groups (AN, BN, EDNOS). However, when the groups were divided into restrictive and binge-purge subtypes, significant differences were found. The AN-BP group had higher CIA scores than the AN-R and EDNOS-R groups. The BN group also had higher CIA scores than the EDNOS-R group. This suggests a greater degree of functional impairment in binge-purge spectrum diagnoses, and particularly in binge-purge type AN. This finding is consistent with the fact that the binge-purge spectrum groups also seemed to have a higher degree of clinical severity than restrictive groups, as measured by the EDE global and subscale scores. The exception to this pattern was the EDE dietary restraint subscale, which was elevated in the AN-BP group alone, relative to all other diagnostic groups. Highly restrictive eating patterns therefore seem to particularly characterise individuals with an AN-BP diagnosis, which may account for their ability to maintain a low BMI (that is BMI < 17.5) despite engaging in episodes of binge eating.

In all diagnostic groups, CIA scores were positively correlated with EDE global scores and subscale scores. The prominent contribution of attitudinal disturbances to poor QoL in ED has recently also been highlighted by Latner and colleagues [38] in a mixed ED outpatient sample.

However, CIA scores were not correlated with other clinical variables such as BMI, illness duration or frequency of bingeing and/or purging behaviours. The exception to this pattern was the AN-BP group, for whom the CIA score was negatively correlated with BMI and with frequency of OBEs, that is for individuals with an AN-BP diagnosis, a higher BMI and more frequent OBEs were associated with reduced impairment. This suggests that, for people with AN-BP, a higher BMI acts as a protective factor, reducing the functional impairment associated with this diagnosis. It is possible that a combined effect of binge-purge behaviours and low BMI accounts for the particularly poor QoL in this group. The relationship between CIA scores and OBE frequency is surprising, given that binge eating is typically experienced by patients as highly distressing and causing significant impairment. One possibility is that calorie consumption during episodes of binge eating helps to mitigate the negative effects of extreme dietary restriction in this group. This finding was however only a statistical trend and requires further exploration to establish whether it can be reliably replicated.

In terms of prognosis and need for treatment, AN is considered to be the most severe ED. The finding that individuals with restrictive EDs, including AN, report less impairment and lower illness severity than other groups seems inconsistent with this view. One possibility is that the EDE and CIA are less sensitive to the symptoms and impairment associated with restrictive EDs. There is some evidence that the EDE is insufficiently sensitive and fails to detect all cases of AN and EDNOS [35], especially for adolescent samples [39]. Previous work also suggests that EDE global scores are somewhat lower in AN than BN patient groups (M_AN_ = 2.65, M_BN_ = 3.07) [31]. The ego-syntonic nature of these disorders may also play a role here; individuals who view their disorder as beneficial or rewarding are likely to report fewer distressing symptoms and less associated impairment [13]. This may be the case for people with restrictive diagnoses, where the resulting weight loss is highly valued and ego-dystonic aspects of the illness may be less apparent. Bingeing and purging behaviours tend to be viewed as highly distressing and therefore individuals with these behaviours may be more aware of the negative impact of their ED. Alternatively, diagnostic differences in treatment seeking behaviour and access to specialist services could account for the lower severity and impairment reported by restrictive patients in this sample.

Clinical understanding suggests that chronic illnesses become more burdensome over time. It is therefore surprising that illness duration was not related to level of impairment in the present study. However, previous research suggests that people with chronic illnesses are motivated to adapt to their symptoms and this creates a complex relationship between illness chronicity and QoL [40]. It is hypothesized that chronic illness produces a change in values and expectations that allows the individual to adapt to the functional impairment associated with his/her disorder – a change known as response shift [41-43]. Response shift could account for the lack of association found between illness chronicity and QoL in this and other studies [11]. It has also been suggested that any relationship between illness chronicity and QoL in EDs may be obscured by fluctuations in symptom severity over time, given the remitting/relapsing nature of many EDs [18].

### Limitations

A limitation of the current study is the use of a treatment-seeking sample. Many individuals with EDs do not seek treatment from specialist services [44-47] and so findings in this sample may not generalise to other groups, such as community samples of people with EDs. Bohn and colleagues [26] note that it is often psychosocial impairment that leads people with EDs to seek help and so we might expect that a treatment-seeking sample will be particularly impaired.

A second limitation is the lack of a measure of comorbid anxiety and depression. High levels of anxiety and depression are common in people with EDs [48] and are likely to cause significant impairment. The CIA’s focus on impairment that is specifically related to ED thoughts and behaviours may limit the impact of anxiety and/or depression on our findings, but we are not able to exclude the possibility that comorbid anxiety and depression significantly affect CIA scores. One previous study in a community sample found a positive correlation between CIA scores and a measure of depression, although depression was not as strong a predictor of CIA score as were EDE-Q scores [36]. This suggests that general distress, including depression and anxiety, may affect CIA responses – this is something that should be explored in future studies by including measures of comorbid anxiety and depression. Finally, duration of illness was determined by the clinical assessment (rather than by research interview).

### Implications for future research

The present study suggests that there are differences in the degree of impairment across diagnostic groups and in particular, important differences between binge-purge and restrictive subtypes. This highlights the importance of accurately characterising ED samples and differentiating these subtypes in future QoL research. It remains unclear why individuals with restrictive EDs appear to have less severe symptoms and less associated impairment than those with binge-purge EDs. Research using a range of QoL measures could help examine the possibility that this effect is due to insensitivity of the CIA and EDE to detect disorder severity and impairment in this group. Self-report measures could also be supplemented with other indices of impairment (e.g. clinician ratings, reports from close others), in order to explore whether individuals with restrictive EDs under-report illness severity and impairment.

Further studies are needed to examine possible predictors of QoL and impairment in EDs. It seems likely that the relationships between clinical variables and impairment may be complex. For example, any link between illness duration and impairment is likely to be complicated by factors such as response shift and symptom fluctuation over time. A longitudinal approach may therefore be necessary to examine this relationship. Other variables may influence impairment in an interactive way. The particularly high degree of impairment in the binge-purge AN group, and the correlation with BMI in this group, suggests that bingeing and/or purging behaviours and BMI may have interactive or additive effects on functional impairment. Further work with large patient groups is needed to explore this possibility.

### Implications for clinicians

Clinicians need to be aware of the high level of functional impairment found across the EDs, as this impairment is an important focus for intervention and may also significantly impact on factors such as readiness to change and motivation. Across ED diagnoses, CIA scores were very high compared to reported population norms [28,36,37], which suggests that individuals with EDs are severely impaired by their disorder and are very aware of the impairment they experience as a result of their ED. A focus on functional impairment in treatment may therefore help to increase motivation and readiness to change by highlighting the benefits of making changes to current eating behaviours. The CIA seems to be particularly appropriate for use in clinical work, as it was developed with input from ED patients [26], and therefore measures impairment in domains experienced as personally relevant to these patients.

## Conclusion

Patients with EDs have very poor QoL compared to the general population, and individuals with EDs characterised by bingeing and/or purging behaviours seem to be particularly impaired. Impairment also seems to increase with illness severity, but does not appear to be related to the frequency of bingeing/purging behaviours, BMI or illness duration. The exception to this pattern is AN-BP patients, for whom decreased BMI is related to greater impairment. This suggests that the presence of bingeing/purging behaviour and low BMI may have interactive effects on impairment. Further work is needed to examine the role of various clinical variables in predicting the level of impairment associated with EDs and possible interactions between these variables.

## Competing interests

The authors declare that they have no competing interests.

## Authors’ contributions

US and HD conceived and designed the study. HD, AO, LS, NS, MK, HB made substantial contributions to acquisition of data. HD analysed and interpreted the data and drafted the manuscript. All authors contributed to revising it critically for important intellectual content; and all authors read and approved the final manuscript.
